# The simple observational critical care studies: estimations by students, nurses, and physicians of in-hospital and 6-month mortality

**DOI:** 10.1186/s13054-021-03809-w

**Published:** 2021-11-15

**Authors:** Eline G. M. Cox, Marisa Onrust, Madelon E. Vos, Wolter Paans, Willem Dieperink, Jacqueline Koeze, Iwan C. C. van der Horst, Renske Wiersema, SICS Study Group & SOCCS Student Team, Tamar van der Aart, Amila Azdahic, Diede Benjamins, Anke Bergsma, Yorick Bergsma, Jeroen Boekema, Vera Braun, Berend Bremer, Jan-Paul de Bruin, Elisa Chen, Chantal Dankfort, Karin Delfsma, Isabel Dieleman, Allysa Dijkstra, Alma Dijkstra, Elsa Doornbos, Ruben Eck, Rebecca van Elst, Dajana Erceg, Koen Geertzen, Chris Govaerts, Dion Groothof, Elisabeth Hagenauw, Xantia Heeres, Bart Hiemstra, Jildou Hoogland, Gerben Horstink, Maike Huizenga, Alexander Irk, Astrid Jansen, Thibault de Jonge, Thomas Kaufmann, Britt Keuning, Eric Keus, Charlotte Koerts, Evelyn van der Kooi, Femia Koopmans, Lisa Koops, Geert Koster, Menno de Leeuw, Lothar Mastenbroek, Ahra Meetsma, Gwen Miedema, Hidde Pelsma, George Pijpstra, Sarah Pruckl, Arlinde Roelofs, Maaike Schagen, Carine Schilte, Kim Selles, Elma Sluiter, Anna Smit, Lou van der Starre, Paul van Stee, Tim Takkenkamp, Melanie Theunis, Leonie Tijsma, Kabir Tombat, Nymke Trouwborst, Fennie Uiterwijk, Nynke van der Veen, Dorje Meilink, Bart Vinke, Erin Anne Visser, Brenda Wiersma, Marleen Wijma, Hinse Wiltingh, Jelle Wolters

**Affiliations:** 1 Department of Critical Care, University Medical Center Groningen, University of Groningen, PO Box 30.001, 9700 RB Groningen, The Netherlands; 2 Department of Anesthesiology, University Medical Center Groningen, University of Groningen, Groningen, The Netherlands; 3 Research Group Nursing Diagnostics, Hanze University of Applied Sciences, Groningen, The Netherlands; 4 Department of Intensive Care Medicine, University Medical Center Maastricht+, University of Maastricht, Maastricht, The Netherlands; 5 Cardiovascular Research Institute Maastricht (CARIM), Maastricht, The Netherlands; 6 Department of Cardiology, Medisch Centrum Leeuwarden, Leeuwarden, The Netherlands

**Keywords:** Critically ill patients, Prognostic, Estimations, Students, Nurses, Physicians, Mortality prediction

## Abstract

**Background:**

Prognostic assessments of the mortality of critically ill patients are frequently performed in daily clinical practice and provide prognostic guidance in treatment decisions. In contrast to several sophisticated tools, prognostic estimations made by healthcare providers are always available and accessible, are performed daily, and might have an additive value to guide clinical decision-making. The aim of this study was to evaluate the accuracy of students’, nurses’, and physicians’ estimations and the association of their combined estimations with in-hospital mortality and 6-month follow-up.

**Methods:**

The Simple Observational Critical Care Studies is a prospective observational single-center study in a tertiary teaching hospital in the Netherlands. All patients acutely admitted to the intensive care unit were included. Within 3 h of admission to the intensive care unit, a medical or nursing student, a nurse, and a physician independently predicted in-hospital and 6-month mortality. Logistic regression was used to assess the associations between predictions and the actual outcome; the area under the receiver operating characteristics (AUROC) was calculated to estimate the discriminative accuracy of the students, nurses, and physicians.

**Results:**

In 827 out of 1,010 patients, in-hospital mortality rates were predicted to be 11%, 15%, and 17% by medical students, nurses, and physicians, respectively. The estimations of students, nurses, and physicians were all associated with in-hospital mortality (OR 5.8, 95% CI [3.7, 9.2], OR 4.7, 95% CI [3.0, 7.3], and OR 7.7 95% CI [4.7, 12.8], respectively). Discriminative accuracy was moderate for all students, nurses, and physicians (between 0.58 and 0.68). When more estimations were of non-survival, the odds of non-survival increased (OR 2.4 95% CI [1.9, 3.1]) per additional estimate, AUROC 0.70 (0.65, 0.76). For 6-month mortality predictions, similar results were observed.

**Conclusions:**

Based on the initial examination, students, nurses, and physicians can only moderately predict in-hospital and 6-month mortality in critically ill patients. Combined estimations led to more accurate predictions and may serve as an example of the benefit of multidisciplinary clinical care and future research efforts.

**Supplementary Information:**

The online version contains supplementary material available at 10.1186/s13054-021-03809-w.

## Background

Patients admitted to intensive care units (ICUs) may suffer from various illnesses and comorbidities. ICU patients are known to have high mortality rates, and survivors may suffer from long-term impairments in both cognitive and overall function, leading to a reduced quality of life [1]. Although all patients admitted to the ICUs are in an acute state of critical illness, they are often remarkably different in terms of characteristics and prognosis. Every day, healthcare providers judge clinical expectations, including possible health outcomes, and provide such information to patients and their relatives. Accurate estimates of survival are essential, as these may influence clinical decisions, especially in resource-limited situations. In an ICU setting, physicians and nurses typically apply a combination of deductive and inductive reasoning, along with intuitive (non-methodical reasoning) analysis. Discussing mortality estimations in an inter-disciplinary setting can therefore offer new insights and improvements in the reasoning process and improved prediction [2, 3].

During the training of physicians and nurses, various theories are used to teach them how to actively combine acquired clinical knowledge, acquired diagnostic experience, and personal intuition. This approach is needed to make as accurate prognostic statements as possible, including statements about the likelihood that the applied treatment will lead to survival [4].

One intriguing study evaluated the discriminative accuracy of physicians and nurses’ estimations of 303 critically ill patients' clinical outcomes and found that the estimations of physicians and nurses added significantly to the discriminative accuracy of existing prediction models [5]. However, the results of this study have not been validated in a sufficiently large population [6], and research in other settings has shown contrasting results [7]. Nevertheless, the estimation would be valuable in the early stages of critical diseases and should be made shortly after ICU admission. Furthermore, the accuracy of estimations can evolve and differ for different healthcare providers [8–10].

The ability of healthcare providers to predict which patients will survive is relevant for clinical decision-making. However, intuition alone may not always lead to correct diagnostic judgments, and both managing this intuition and clinical experience are prerequisites for reliable estimates of the outcome. The estimations of experienced physicians and nurses may be substantially more accurate than those of students. Whether this assumption is correct is unknown, however. This study aimed to explore the accuracy of students’, nurses’, and physicians’ estimations of mortality and to evaluate the association of combined estimations by students, nurses, and physicians with the outcome.

## Methods

### Design and setting

The Simple Observational Critical Care Studies (SOCCS, NCT03553069) is a single-center, prospective observational study designed to evaluate the diagnostic and prognostic value of clinical examination on admission and serves as the first time point for repeated clinical examination in critically ill patients as part of the Simple Intensive Care Studies II (SICS-II, NCT03577405) study [11, 12]. The local institutional review board approved the study (2018/203).

### Participants

This study included all acutely admitted critically ill patients over 18 years of age, with an expected stay of at least 24 h. Patients were excluded if they had previously been included in this study (e.g., in cases of readmission), if strict isolation rules limited access, or if no informed consent was obtained.

### Variables

Patient characteristics were registered, including age, gender and body mass index were registered at admission, and outcome was estimated according to our protocol [11]. All acutely admitted patients were included as soon as possible after acute admission during the day- and nighttime, with a maximum of 3 h after ICU admission. A selected team of about thirty medical and nursing students was available for shifts to support research in our ICU voluntarily. All medical and nursing students had completed at least one year of medical or nursing school and were trained to perform a structured one-time clinical examination. This type of examination was familiar to all students from their medical and nursing education, including skills.

When a patient was acutely admitted to the ICU, the SOCCS team was called by the study coordinator (daytime) or physician (nighttime). The student first performed a structured clinical examination, assessing basic vitals and signs of shock. Subsequently, the medical student, an ICU nurse, and a physician independently answered the following questions: (1) "Do you think the patient will survive this hospital admission?"; (2) "Do you think the patient will be alive in 6 months?" [13, 14]. The physicians and nurses received no additional training but were all informed about the study. Clinical risk scores were calculated after the termination of the study and were not available for the researcher at the time of estimation. The students were not involved in patient care and were instructed not to share their findings with the attending physicians and nurses. Instead, mortality data were gathered from the electronic health records.

### Statistics

Continuous variables were reported as means (with standard deviations) or medians (with interquartile ranges (IQRs)) depending on distributions. Categorical data were presented in proportions. Associations were calculated as odds ratios with 95% confidence intervals (CI). Student's t tests, Mann–Whitney *U* tests, or the Chi-square tests were used as appropriate. In addition, the clinical characteristics of patients were explored.

The univariate association between single estimations and in-hospital mortality and the association of cumulative estimations and in-hospital mortality were explored using univariate logistic regression analysis. To test for marginal homogeneity of estimations between groups, a McNemar test was performed. Area under the receiving operator characteristic (AUROC) was calculated for each estimation to assess discriminative accuracy. Values of *p* <0.05 were considered statistically significant. Analyses were performed using Stata version 16 (StataCorp, College Station, TX, USA).

## Results

**Fig. 1:**
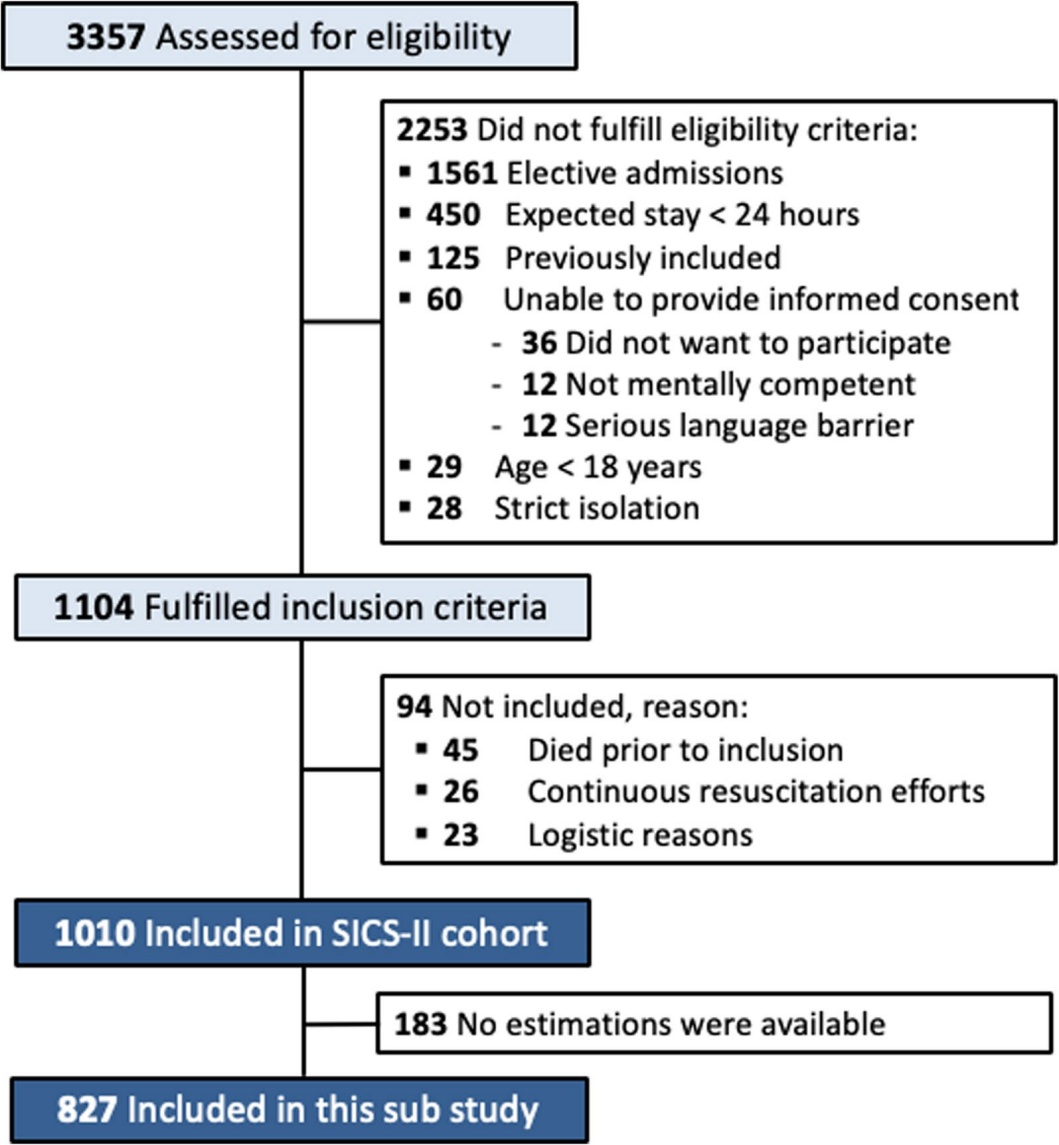
Flowchart of study inclusion

Between May 14, 2018, and July 10, 2019, a total of 3357 ICU admissions were assessed for eligibility. In total, 1,104 patients fulfilled the inclusion criteria for clinical examination. Data were not obtained for 94 patients: 45 patients died before inclusion, continuous resuscitation efforts were made for 26 patients, and there was no access to 23 patients due to logistic reasons. In total, 1,010 patients were included in the SICS-II cohort. For 183 patients (18%), estimations were not available, resulting in 827 patients (82%) being included in the SOCCS cohort (Fig. 1; Table 1). Median time from ICU admission to clinical examination was 1.8 h (IQR 1.1–2.7). In total, 178 patients (22%) died during the hospital stay. By the 6-month follow-up, 238 patients (29%) had died. Ten patients were lost to follow-up.

**Table 1:** Clinical characteristics of the SOCCS cohort

Variable	Patients in SOCCS *N* = 827
Age, years (SD)	60 (16)
Sex, male (%)	506 (61)
BMI, kg/m^2^ (SD)	26 (5)
Diabetes mellitus, n (%)	147 (18)
Liver cirrhosis, n (%)	39 (5)
Mechanical ventilation at inclusion, *n* (%)	428 (52)
SAPS II, score (SD)^a^	41 (17)
APACHE IV, score (SD)^a^	70 (31)
*Central circulation*	
Respiratory rate, per minute (SD)	18 (6)
Heart rate, beats per minute (SD)	90 (23)
Systolic blood pressure, mmHg (SD)	118 (28)
Diastolic blood pressure, mmHg (SD)	62 (13)
Mean arterial pressure, mmHg (SD)	81 (21)
Use of vasopressors at inclusion, *n* (%)	376 (45)
*Organ perfusion*	
Consciousness	
Alert, *n* (%)	548 (67)
Reacting to voice, *n* (%)	102 (12)
Reacting to pain, *n* (%)	17 (2)
Unresponsive, *n* (%)	152 (19)
Central temperature, °C (SD)	37 (1)
Temperature dorsum foot, °C (SD)	30 (3)
Cold extremities, subjective, *n* (%)	252 (32)
Capillary refill time sternum, *s* (SD)	3 (1)
Capillary refill time knee, *s* (SD)	3 (2)
*Skin mottling severity*^b^	
Mild (0–1)	638 (9%)
Moderate (2–3)	72 (10%)
Severe (4–5)	5 (< 1%)

SD, standard deviation; BMI, body mass index; APACHE IV, acute physiology, and chronic health evaluation

^a^Clinical risk scores were calculated after the termination of the study and were not available for the researcher at the time of estimation

^b^Mottling was scored according to Ait-Ouffella et al. [35]

### Students’, nurses’, and physicians’ estimations

Estimations concerning in-hospital mortality were obtained from medical students in all cases, from the nurses in 709 cases (86%), and from the physicians in 507 cases (61%). There were no significant differences between the characteristics and severity of the disease of patients who had and patients who did not have an estimation by the physician (Additional file 1: Table S1).

### In-hospital mortality

Medical and nursing students predicted in-hospital mortality of 11%, nurses 15%, and physicians 17%. There was a fair agreement for all students, nurses, and physicians estimating in-hospital mortality (range 87.4–90.8%) (Table 2).

**Table 2:** Agreement between students, nurses, and physicians for estimating in-hospital mortality

	% agreement	Kappa (95% CI)	McNemar's test (*p* value)
Nurse versus student	90.8	0.59 (0.50–0.68)	< 0.01
Physician versus student	87.4	0.46 (0.35–0.57)	< 0.01
Physician versus nurse	90.2	0.62 (0.52–0.72)	< 0.01

Estimations of all students, nurses, and physicians were available in 481 cases, and all were correct in 71% of cases. There were no significant differences in the proportions of correct estimates between groups (all *p* values > 0.2). The estimations of students, nurses, and physicians were all associated with in-hospital mortality (OR 5.8, CI [3.6, 9.4], OR 4.7, [2.9, 7.5], and OR 7.7 [4.5, 13.2], respectively). Discriminative accuracy was moderate for students, nurses, and physicians, with AUROC ranging from 0.61 to 0.68. When more estimates were of non-survival, odds of non-survival increased (OR 2.4, CI [1.9, 3.1] per additional estimate, AUROC 0.70 CI [0.65, 0.76] (Table 3).

**Table 3:** Univariate associations between estimations and in-hospital mortality

	*N*	OR	95% CI	AUROC (95% CI)
Students estimation	827	5.8	3.6–9.4	0.61 (0.57–0.64)
Nurses estimation	709	4.7	2.9–7.5	0.62 (0.58–0.66)
Physicians estimation	507	7.7	4.5–13.2	0.68 (0.63–0.73)
Cumulative estimation	481	2.4	1.9–3.1	0.70 (0.65–0.76)

### Mortality at 6 months

Six-month mortality was predicted to be 18% by students, 20% by nurses, and 21% by physicians. The estimations of students, nurses, and physicians were all associated with 6-month mortality (OR 4.4, CI [3.1, 6.4], OR 6.3, CI [4.2, 9.4] and OR 7.1, CI [4.4, 11.2], respectively), and clinical characteristics were different when mortality was estimated (Additional file 2: Table S2a-c). When more estimates were of non-survival, odds of non-survival increased (OR 2.5, CI [2.0, 3.1]) per additional estimate). In total, estimations of all students, nurses, and physicians for 6-month mortality were available in 479 cases. There were 41 (9%) patients for whom all students, nurses, and physicians predicted non-survival at 6 months. These patients more often received vasoactive medication, had a lower temperature, and had higher APACHE IV and SAPS II scores (Additional file 3: Table S3). Discriminative accuracy was moderate for all students, nurses, and physicians, with AUROC ranging from 0.62 to 0.68, but was fair when estimations were combined (0.72, CI [0.67, 0.76]) (Table 4).

**Table 4:** Univariate associations between estimations and six-month mortality

	*N*	OR	95% CI	AUROC (95% CI)
Students estimation	819	4.4	3.1–6.4	0.62 (0.59–0.66)
Nurses estimation	699	6.3	4.2–9.4	0.66 (0.62–0.69)
Physicians estimation	503	7.1	4.4–11.2	0.68 (0.63–0.72)
Cumulative estimation	475	2.5	2.0–3.1	0.72 (0.67–0.76)

## Discussion

In this study, the estimations of medical and nursing students, nurses, and physicians of both in-hospital and 6-month mortality within 3 h after admission to the ICU were found to have moderate discriminative accuracy. In addition, the variation in accuracy between students, nurses, and physicians was not statistically different. The most valuable observation was that, in case where more students, nurses, and physicians estimated non-survival, both the risk of death and the discriminative accuracy were significantly higher, suggesting the benefits of multidisciplinary clinical care and prognostication.

Several studies have evaluated the accuracy of physicians and nurses’ estimations of clinical outcomes in critically ill patients [5, 15–19]. Overall, these studies show an additional value of physicians and nurses’ estimations compared or added to existing clinical prediction models. Below, we present the previous studies most relevant to the current study. Detsky et al. investigated the discriminative accuracy of both nurses' and physicians’ estimations and showed that, of multiple outcomes estimated, nurses could best estimate in-hospital mortality in critically ill patients [5]. In our study, physicians were most accurate in estimating in-hospital mortality, followed by students and nurses. No previous study has evaluated the accuracy of medical students’ estimations of patient mortality. Buehler et al. showed that the overall accuracy of predictions made by ICU physicians was moderate [20]. However, their study was a literature review comparing several observational studies that were limited to physicians.

We are unaware of any studies investigating the cumulative estimation of multiple healthcare providers considering mortality. However, one study showed that predictions considering ineffective treatment were more valuable when based on collaborative decision-making [21]. Another study focusing on detecting sepsis showed that the chance of severe sepsis was highest when estimated by two healthcare providers [22].

The most significant difference between our study and previous research efforts is that we intended to assess patients at the earliest ICU admission stage. In previous studies, the estimations were made within 24 h after ICU admission, leaving time for interventions and treatment responses that which might influence outcome predictions, and were mainly made by fewer physicians [23]. Treatment decisions and interventions influence prognostication only minimally in the first few hours after admission. Thereafter, many other factors, such as deterioration or improvement of organ function, logically influence the (prediction of the) outcome. To eventually guide clinical decision-making, it is essential to estimate mortality as soon as possible after, or even at, ICU admission to trigger reconsideration of ICU admittance and treatment restrictions.

The moderate accuracy of clinical predictions by individuals has been well established [24]. It is assumed that many supportive technical innovations in clinical decision-making will exponentially become available [25]. The challenge is to find and especially use the best possible algorithm to predict patient outcomes [26]. Nevertheless, healthcare providers can take into account improbable events and take variables that are currently unavailable for algorithms. It remains essential to keep an eye on the patient at the bedside, as previous studies have shown that often simple variables can predict adverse outcomes [27, 28]. Furthermore, the healthcare providers' “gut feeling,” or “clinical gestalt,” will probably never be fully understood but may be of crucial additive value. For example, Ferreyro et al. investigated physician- and patient-level factors influencing physicians' predictions of 6-month mortality in critically ill patients based on variables available at admission [29]. The authors found that older age, malignancy, and higher APACHE III scores were associated with the physicians’ predictions [29]. Although we did not investigate students, nurses, and physicians factors, we report similar observations for patient-level factors such as the SAPS II and APACHE IV score, the use of vasopressors, prolonged capillary refill time, and lower level of consciousness in our study. Using data from studies like from Ferreyro et al. or our study using Bayesian network analysis may further unravel how, why, and based on what variables healthcare providers make their predictions [30, 31]. Eventually, insight into the drivers of clinical predictions could be valuable for developing and evaluating decision algorithms and may inform future healthcare providers' education [32].

### Implications and generalizability

This was a single-center study; however, outcome estimations are free, available worldwide, and do not seem to depend on ICU facilities. Our study design is widely applicable, and collaboration with other centers and other ICU departments might increase generalizability. It is important to note that these findings result from univariate analysis, as mortality prediction models are widely available [33], and developing a new one lies beyond the scope of this study. Currently, we do not know which factors help healthcare providers make their “educated guess.” Future research should focus on evaluating the conditional dependencies between the estimations and variables obtained from clinical examination to assess how healthcare providers reach their estimations (e.g., using Bayesian networks) [30]. This may lead to a better understanding of how these factors influence clinical decision-making and improve outcome prediction [34]. In the current study, we included nurses and medical students and their predictions, which led to improved collaborative mortality prediction. We showed that multidisciplinary research, including various healthcare providers with different experience levels, can lead to a successful observational study. We have learned that this experience—both participating in research and learning to perform a structured clinical examination of a critically ill patient—has benefitted the junior researchers involved during their medical or research career. In addition, it would be interesting to assess the rate of withdrawal of care during the ICU stay in future studies.

## Limitations

Several limitations of this study must be acknowledged. First, students’, nurses', and physicians’ estimations are subjective and may be influenced by personal experience, beliefs, or specialty training. Second, unlike other studies, we did not report any characteristics of the students, nurses, and physicians, who provided the estimations of mortality [5, 23]. Furthermore, we reported the estimations based on a binary scale, using “Yes” and “No.” Physicians may be more likely to make mortality predictions on a continuous scale. Using a rating scale based on likelihood and providing more characteristics of the healthcare providers could have helped further characterize our results. Third, the results of the estimations of the nurses and physicians need to be interpreted carefully since the a priori chance of a good outcome is, in general, relatively high and might therefore influence the overall accuracy. Moreover, the students were instructed to complete their assessments first, and the physicians and nurses were also instructed to let the student make their estimates first. However, the students were not completely blinded to the estimations of the physicians and nurses, which could have influenced their estimations. Furthermore, there might have been a selection bias due to the relatively high number of missing estimations by physicians. These estimations, and those of others, may be missing because the physician or nurse was absent or occupied at the time students made their estimations. However, there was no significant clinical difference between patients with and without an estimation of mortality by the physician, and as we included a large cohort of unselected patients, we deem it unlikely that this could have influenced our results.

## Conclusion

Based on the initial examination, students, nurses, and physicians can only moderately predict in-hospital and 6-month mortality in critically ill patients. Combined estimations led to more accurate predictions and may serve as an example of the benefit of multidisciplinary clinical care and future research efforts.

## Supplementary Information

**Additional file 1: Table S1**. Clinical characteristics of patients with and without the physicians' estimation.

**Additional file 2: Table S2a**. Clinical characteristics of patients when the student estimated survival or non-survival. **Table S2b**. Clinical characteristics of patients when the nurse estimated survival or non-survival. **Table S2c**. Clinical characteristics of patients when the physician estimated survival or non-survival.

**Additional file 3: Table S3**. Clinical characteristics of patients when all students, nurses, and physicians estimated non-survival.

## Abbreviations

ICU: Intensive Care Unit
SICS-I: Simple Intensive Care Studies-I
SICS-II: Simple Intensive Care Studies-II
SOCCS: Simple Observational Critical Care Studies
UMCG: University Medical Center Groningen
APACHE IV: Acute Physiology and Chronic Health Evaluation
AUROC: Area under the receiver operating curve
CI: Confidence interval
OR: Odds ratio

## Declarations

### Availability of data and materials

The datasets used and/or analyzed during the current study are available from the corresponding author on reasonable request.
