# Supplementary material for: The simple observational critical care studies: estimations by students, nurses, and physicians of in-hospital and 6-month mortality
Source: Crit Care. 2021 Nov 15;25:393. doi: 10.1186/s13054-021-03809-w (PMC8591867; doi:10.1186/s13054-021-03809-w)
Supplement: Supplementary file 1 — Additional file 1: Table S1. Clinical characteristics of patients with and without the physicians' estimation. [file 13054_2021_3809_MOESM1_ESM.docx]

**eTable 1. Clinical characteristics of patients with and without the physicians' estimation**

| Variable | *With physicians' estimation*  N = 507 | *Without physicians' estimation*  N = 320 | *p-value* |
| --- | --- | --- | --- |
| Age, years (SD)  Sex, male (%)  BMI, kg/m^2^ (SD)  Diabetes mellitus, n (%)  Liver cirrhosis, n (%)  Mechanical ventilation at inclusion, n (%)  SAPS II, score (SD)  APACHE IV, score (SD)  Central circulation | 60 (15)  304 (60)  26 (5)  93 (18)  24 (5)  264 (52)  41 (18)  70 (33) | 61 (16)  202 (63)  26 (5)  54 (17)  15 (5)  164 (51)  41 (15)  69 (28) | 0.17  0.36  0.78  0.59  0.97  0.82  0.98  0.48 |
| Respiratory rate, per minute (SD) | 18 (6) | 19 (6) | 0.51 |
| Heart rate, beats per minute (SD) | 89 (23) | 92 (23) | 0.063 |
| Systolic blood pressure, mmHg (SD) | 118 (28) | 119 (27) | 0.81 |
| Diastolic blood pressure, mmHg (SD)  Mean arterial pressure, mmHg (SD) | 62 (14)  81 (21) | 63 (13)  82 (21) | 0.28  0.58 |
| Use of vasopressors at inclusion, n (%)  Organ perfusion  *Consciousness* | 229 (45) | 147 (46) | 0.83  0.88 |
| Alert, n (%)  Reacting to voice, n (%)  Reacting to pain, n (%)  Unresponsive, n (%)  Central temperature, °C (SD)  Temperature dorsum foot, °C (SD)  Cold extremities, subjective, n (%)  Capillary refill time sternum, s (SD)  Capillary refill time knee, s (SD)  Skin mottling severity^a^   - Mild (0-1) - Moderate (2-3) - Severe (4-5) | 331 (66)  63 (13)  11 (2)  97 (19)  37 (1)  30 (3)  160 (32)  3 (1)  3 (2)  419 (83%)  51 (10%)  3 (<1%) | 217 (68)  39 (12)  6 (2)  55 (17)  37 (1)  30 (3)  92 (33)  3 (1)  3 (2)  219 (68%)  21 (7%)  2 (<1%) | 0.45  0.67  0.88  0.34  0.49  0.16 |

Abbreviations: SD = standard deviation, BMI = Body Mass Index, APACHE IV = acute physiology, and chronic health evaluation.

^a^ Mottling was scored according to Ait-Ouffella et al. ^1^

1. Ait-Oufella H, Lemoinne S, Boelle PY, et al. Mottling score predicts survival in septic shock. *Intensive Care Med*. 2011;37(5):801-807.
