# Supplementary material for: The simple observational critical care studies: estimations by students, nurses, and physicians of in-hospital and 6-month mortality
Source: Crit Care. 2021 Nov 15;25:393. doi: 10.1186/s13054-021-03809-w (PMC8591867; doi:10.1186/s13054-021-03809-w)
Supplement: Supplementary file 2 — Additional file 2: Table S2a. Clinical characteristics of patients when the student estimated survival or non-survival. Table S2b. Clinical characteristics of patients when the nurse estimated survival or non-survival. Table S2c. Clinical characteristics of patients when the physician estimated survival or non-survival. [file 13054_2021_3809_MOESM2_ESM.docx]

**eTable 2a. Clinical characteristics of patients when the student estimated survival or non-survival**

| Variable | *Estimated survival*  N = 675 | *Estimated non-survival*  N = 152 | *p-value* |
| --- | --- | --- | --- |
| Age, years (SD)  Sex, male (%)  BMI, kg/m^2^ (SD)  Diabetes mellitus, n (%)  Liver cirrhosis, n (%)  Mechanical ventilation at inclusion, n (%)  SAPS II, score (SD)  APACHE IV, score (SD)  Central circulation | 59 (16)  415 (61)  26 (5)  113 (17)  29 (4)  329 (49)  38 (15)  63 (27) | 65 (13)  91 (60)  25 (4)  34 (22)  10 (7)  99 (65)  56 (17)  97 (33) | <0.001  0.71  0.067  0.10  0.23  <0.001  <0.001  <0.001 |
| Respiratory rate, per minute (SD) | 18 (6) | 20 (6) | 0.004 |
| Heart rate, beats per minute (SD) | 89 (22) | 95 (25) | 0.001 |
| Systolic blood pressure, mmHg (SD) | 120 (28) | 112 (27) | 0.002 |
| Diastolic blood pressure, mmHg (SD)  Mean arterial pressure, mmHg (SD) | 63 (13)  82 (21) | 60 (13)  77 (18) | 0.008  0.010 |
| Use of vasopressors at inclusion, n (%)  Organ perfusion  *Consciousness* | 272 (40) | 104 (68) | <0.001  <0.001 |
| Alert, n (%)  Reacting to voice, n (%)  Reacting to pain, n (%)  Unresponsive, n (%)  Central temperature, °C (SD)  Temperature dorsum foot, °C (SD)  Cold extremities, subjective, n (%)  Capillary refill time sternum, s (SD)  Capillary refill time knee, s (SD)  Skin mottling severity^a^   - Mild (0-1) - Moderate (2-3) - Severe (4-5) | 472 (71)  89 (13)  13 (2)  94 (14)  37 (1)  30 (3)  193 (30)  3 (1)  3 (2)  530 (90%)  53 (9%)  4 (1%) | 76 (50)  13 (9)  4 (3)  58 (38)  36 (2)  30 (4)  59 (41)  3 (2)  4 (3)  108 (84%)  19 (15%)  1 (1%) | 0.031  0.94  0.015  <0.001  <0.001  <0.001 |

Abbreviations: SD = standard deviation, BMI = Body Mass Index, APACHE IV = acute physiology, and chronic health evaluation.

^a^ Mottling was scored according to Ait-Ouffella et al. ^1^

**eTable 2b. Clinical characteristics of patients when the nurse estimated survival or non-survival**

| Variable | *Estimated survival*  N = 568 | *Estimated non-survival*  N = 139 | *p-value* |
| --- | --- | --- | --- |
| Age, years (SD)  Sex, male (%)  BMI, kg/m^2^ (SD)  Diabetes mellitus, n (%)  Liver cirrhosis, n (%)  Mechanical ventilation at inclusion, n (%)  SAPS II, score (SD)  APACHE IV, score (SD)  Central circulation | 59 (16)  346 (61)  26 (5)  97 (17)  28 (5)  282 (50)  38 (15)  62 (26) | 64 (14)  86 (62)  26 (5)  28 (20)  6 (4)  89 (64)  54 (17)  89 (64) | 0.003  0.84  0.56  0.40  0.77  0.002  <0.001  <0.001 |
| Respiratory rate, per minute (SD) | 18 (6) | 20 (6) | <0.001 |
| Heart rate, beats per minute (SD) | 87 (21) | 95 (23) | <0.001 |
| Systolic blood pressure, mmHg (SD) | 119 (28) | 117 (27) | 0.47 |
| Diastolic blood pressure, mmHg (SD)  Mean arterial pressure, mmHg (SD) | 62 (13)  81 (20) | 61 (13)  79 (15) | 0.45  0.17 |
| Use of vasopressors at inclusion, n (%)  Organ perfusion  *Consciousness* | 233 (41) | 88 (63) | <0.001  <0.001 |
| Alert, n (%)  Reacting to voice, n (%)  Reacting to pain, n (%)  Unresponsive, n (%)  Central temperature, °C (SD)  Temperature dorsum foot, °C (SD)  Cold extremities, subjective, n (%)  Capillary refill time sternum, s (SD)  Capillary refill time knee, s (SD)  Skin mottling severity^a^   - Mild (0-1) - Moderate (2-3) - Severe (4-5) | 402 (71)  77 (14)  13 (2)  71 (13)  37 (1)  30 (3)  167 (30)  3 (1)  3 (2)  476 (91%)  45 (9%)  2 (<1%) | 68 (50)  12 (9)  2 (1)  55 (40)  37 (2)  30 (4)  57 (41)  3 (2)  4 (2)  105 (82%)  21 (16%)  2 (2%) | 0.11  0.88  0.009  <0.001  0.005  0.002 |

Abbreviations: SD = standard deviation, BMI = Body Mass Index, APACHE IV = acute physiology, and chronic health evaluation.

^a^ Mottling was scored according to Ait-Ouffella et al. ^1^

**eTable 2c. Clinical characteristics of patients when the physician estimated survival or non-survival**

| Variable | *Estimated survival*  N = 400 | *Estimated non-survival*  N = 107 | *p-value* |
| --- | --- | --- | --- |
| Age, years (SD)  Sex, male (%)  BMI, kg/m^2^ (SD)  Diabetes mellitus, n (%)  Liver cirrhosis, n (%)  Mechanical ventilation at inclusion, n (%)  SAPS II, score (SD)  APACHE IV, score (SD)  Central circulation | 59 (16)  233 (58)  26 (5)  73 (18)  17 (4)  198 (50)  38 (16)  62 (27) | 61 (16)  71 (66)  27 (5)  20 (19)  7 (7)  66 (62)  55 (18)  100 (34) | 0.004  0.13  0.22  0.92  0.30  0.025  <0.001  <0.001 |
| Respiratory rate, per minute (SD) | 18 (6) | 19 (7) | 0.024 |
| Heart rate, beats per minute (SD) | 88 (22) | 92 (26) | 0.047 |
| Systolic blood pressure, mmHg (SD) | 119 (29) | 114 (26) | 0.11 |
| Diastolic blood pressure, mmHg (SD)  Mean arterial pressure, mmHg (SD) | 62 (14)  81 (21) | 61 (13)  79 (19) | 0.34  0.19 |
| Use of vasopressors at inclusion, n (%)  Organ perfusion  *Consciousness* | 161 (40) | 67 (63) | <0.001  <0.001 |
| Alert, n (%)  Reacting to voice, n (%)  Reacting to pain, n (%)  Unresponsive, n (%)  Central temperature, °C (SD)  Temperature dorsum foot, °C (SD)  Cold extremities, subjective, n (%)  Capillary refill time sternum, s (SD)  Capillary refill time knee, s (SD)  Skin mottling severity^a^   - Mild (0-1) - Moderate (2-3) - Severe (4-5) | 277 (70)  58 (15)  11 (3)  51 (13)  37 (1)  30 (3)  113 (29)  3 (1)  3 (2)  338 (91%)  32 (9%)  2 (<1%) | 54 (51)  5 (5)  0 (0)  46 (44)  36 (2)  30 (4)  47 (45)  3 (1)  4 (2)  81 (81%)  19 (19%)  21 (1%) | 0.020  0.84  0.002  <0.001  <0.001  0.005 |

Abbreviations: SD = standard deviation, BMI = Body Mass Index, APACHE IV = acute physiology, and chronic health evaluation.

^a^ Mottling was scored according to Ait-Ouffella et al. ^1^

**Reference**

1. Ait-Oufella H, Lemoinne S, Boelle PY, et al. Mottling score predicts survival in septic shock. *Intensive Care Med*. 2011;37(5):801-807.
