# Supplementary material for: The simple observational critical care studies: estimations by students, nurses, and physicians of in-hospital and 6-month mortality
Source: Crit Care. 2021 Nov 15;25:393. doi: 10.1186/s13054-021-03809-w (PMC8591867; doi:10.1186/s13054-021-03809-w)
Supplement: Supplementary file 3 — Additional file 3: Table S3. Clinical characteristics of patients when all students, nurses, and physicians estimated non-survival. [file 13054_2021_3809_MOESM3_ESM.docx]

**eTable 3. Clinical characteristics of patients when all students, nurses, and physicians estimated non-survival**

| Variable | *Survival*  N = 438 | *Non-survival*  N = 41 | *p-value* |
| --- | --- | --- | --- |
| Age, years (SD)  Sex, male (%)  BMI, kg/m^2^ (SD)  Diabetes mellitus, n (%)  Liver cirrhosis, n (%)  Mechanical ventilation at inclusion, n (%)  SAPS II, score (SD)  APACHE IV, score (SD)  Central circulation | 60 (15)  260 (59)  26 (5)  77 (18)  21 (5)  219 (50)  39 (17)  66 (29) | 65 (12)  29 (71)  26 (5)  10 (24)  3 (7)  31 (76)  60 (18)  108 (37) | 0.029  0.15  0.94  0.28  0.49  0.002  <0.001  <0.001 |
| Respiratory rate, per minute (SD) | 18 (6) | 20 (6) | 0.044 |
| Heart rate, beats per minute (SD) | 88 (23) | 93 (26) | 0.18 |
| Systolic blood pressure, mmHg (SD) | 119 (29) | 113 (22) | 0.19 |
| Diastolic blood pressure, mmHg (SD)  Mean arterial pressure, mmHg (SD) | 62 (14)  81 (21) | 61 (13)  78 (15) | 0.79  0.31 |
| Use of vasopressors at inclusion, n (%)  Organ perfusion  *Consciousness* | 181 (41) | 33 (80) | <0.001  <0.001 |
| Alert, n (%)  Reacting to voice, n (%)  Reacting to pain, n (%)  Unresponsive, n (%)  Central temperature, °C (SD)  Temperature dorsum foot, °C (SD)  Cold extremities, subjective, n (%)  Capillary refill time sternum, s (SD)  Capillary refill time knee, s (SD)  Skin mottling severity^a^   - Mild (0-1) - Moderate (2-3) - Severe (4-5) | 295 (68)  57 (13)  11 (3)  70 (16)  37 (1)  30 (3)  134 (31)  3 (1)  3 (2)  365 (89%)  43 (10%)  2 (<1%) | 18 (44)  1 (2)  0 (0)  22 (54)  36 (2)  30 (3)  20 (50)  3 (1)  4 (2)  32 (87%)  5 (13%)  0 (0%) | <0.001  0.77  0.013  0.004  0.043  0.079 |

Abbreviations: SD = standard deviation, BMI = Body Mass Index, APACHE IV = acute physiology, and chronic health evaluation.

^a^ Mottling was scored according to Ait-Ouffella et al. ^1^

1. Ait-Oufella H, Lemoinne S, Boelle PY, et al. Mottling score predicts survival in septic shock. *Intensive Care Med*. 2011;37(5):801-807.
